# Interactive association of chronic illness and food insecurity with emergency department utilization among school‐age children in the United States: A cross‐sectional study

**DOI:** 10.1002/hsr2.1123

**Published:** 2023-02-20

**Authors:** Farheen Ghani, Hao Wang, Sydney E. Manning, Usha Sambamoorthi

**Affiliations:** ^1^ Department of Pharmacotherapy, College of Pharmacy University of North Texas Health Science Center Fort Worth Texas USA; ^2^ Department of Emergency Medicine JPS Health Network Fort Worth Texas USA; ^3^ AcornAI Medidata Solution Boston Massachusetts USA

**Keywords:** chronic conditions, ED utilization, food insecurity, MEPS, school‐age population

## Abstract

**Background and Aims:**

Food insecurity combined with chronic disease conditions is a risk factor for Emergency Department (ED) utilization, an indicator of poor quality of care. However, such an association is not certain among school‐age children with chronic conditions. Therefore, we aim to determine the association of food insecurity, chronic conditions, and ED utilization among school‐age children in the United States.

**Methods:**

We analyzed the data from the 2017 Medical expenditure panel survey (MEPS) among children aged 6–17 years (*N* = 5518). MEPS data was released electronically by the Agency for Healthcare Research and Quality (AHRQ). We identified four groups of school‐age children based on the presence of food security and chronic conditions: 1) with food insecurity and chronic conditions; 2) no food insecurity and chronic conditions; 3) with food insecurity and no chronic conditions; and 4) no food insecurity and no chronic conditions. We compared ED utilization among these four groups using incidence rate ratios (IRR) after adjusting children's age, sex, race and ethnicity, household income, insurance coverage, obesity, and geographic region using count data model, specifically multivariable Poison regression. We used SAS 9.4 and STATA 14.2 for all the data analyses.

**Results:**

There were unweighted 5518 school‐age children who represented weighted 50,479,419 school‐age children in the final analysis. Overall, 6.0% had food insecurity with chronic conditions. These children had higher ED utilization (19.7%) than the other three groups (13.3%, 8.8%, and 7.2%, *p* < 0.001). The adjusted IRR of ED utilization among school‐age children with food insecurity and chronic conditions was 1.90 (95% confidence interval 1.20–3.01, *p* = 0.007) compared with those with food security and chronic conditions.

**Conclusion:**

One in 16 school‐age children has both food insecurity and chronic conditions. Food insecurity was positively associated with frequent ED visits in the presence of chronic conditions. Therefore, addressing food insecurity may reduce the risk of ED visits.

## 1 INTRODUCTION

Food insecurity is defined as “inability to acquire adequate food for one or more household members because they had insufficient money and other resources for food.”^1^ In 2020, 7.6% (2.9 million) households in the United States with children reported food insecurity. The prevalence of food insecurity in households with children increased in 2020 to 14.8% from 13.6% in 2019.^1^ In addition, there are disparities in the prevalence of food insecurity by race and ethnicity, disability status, and urbanicity.^2^ Studies have demonstrated that children living in households with food insecurity are at risk for poor physical, emotional, and developmental outcomes.^3^, ^4^, ^5^ There is robust evidence of the positive relationship of food insecurity with adverse health outcomes.^6^

Food insecurity can lead to high healthcare utilization including preventable hospitalizations,^7^ emergency department (ED) visits,^8^ and high healthcare costs.^9^, ^10^, ^11^ Using 2016 data from the Medical Expenditure Panel Survey (MEPS), which contained information about sociodemographics, health needs, healthcare service uses, and healthcare cost, authors found that children living in households with food insecurity were more likely to have ED use.^12^ However, this study did not analyze the interactive association of chronic conditions and food insecurity linked to ED utilization. It is essential to examine ED visits because such visits are costly^13^ and may represent poor management of chronic conditions,^14^ poor quality of care,^15^ lack of access to emergent care,^14^ and maybe preventable with appropriate primary care.^16^

Food insecurity is reported to be common in pediatric EDs.^17^, ^18^ In a study of 4674 children seen at a general pediatric clinic between 2017 and 2021, food insecurity was reported to be associated with increased ED utilization and hospitalizations.^19^ However, these studies were conducted only among those who visited ED. Given that children with chronic conditions are more likely to use ED than those without chronic conditions,^20^ examining the excess burden of food insecurity on ED use among children with chronic conditions is important. However, such studies are sparse. Determining such an association between food insecurity, chronic disease conditions, and ED utilization among pediatric populations could serve as a foundation to assist families in obtaining services to prevent food insecurity, improve healthcare outcomes, and eventually reduce healthcare costs. Therefore, the primary objective of this paper was to determine the interactive association of chronic illness and food insecurity with ED utilization among school‐age children using well‐established and validated nationally representative survey data in the United States of America.

## 2 METHODS

### 2.1 Study design and data source

This study adopted a cross‐sectional study design. Data were obtained from the 2017 MEPS (i.e., full‐year consolidated data, medical condition data, released on March 4, 2020). MEPS is a nationally representative survey of noninstitutionalized civilian households administered by the Agency for Healthcare Research and Quality (AHRQ). The survey collects information on healthcare services utilization and costs, demographic characteristics, social determinants of health (SDoH, such as education, employment, and income of households), and health status. Food security information was collected in a separate module in 2017. We limited our study to data collected in the calendar year 2017. As MEPS is a publicly available data set with deidentified data, this study was submitted to regional institution review board and approved as a nonhuman subject research project.

### 2.2 Participants

The study included school‐age children 6–17 years of age (*N* = 5691). Among these children, 173 did not have data on food insecurity. The final study sample included 5518 (2809 males with weighted 51.2% and 2709 females with weighted 48.8%) children that represented 50,479,419 children aged from 6 to 17 years.

### 2.3 Measures

#### 2.3.1 Primary outcome measurement

Our primary outcome was the number of ED visits reported for the survey year. The number of ED visits ranged from 0 to 9 in our analytic sample. The MEPS household file contains utilization and expenditure data from the household self‐reports and the medical providers. The medical provider component (MPC) data are not publicly available and are used for editing and imputing household file data.^21^ MPC data include ED visits that may or may not have resulted in an inpatient stay.^22^

#### 2.3.2 Key Explanatory Variable

Food insecurity and chronic condition categories.

##### Presence of chronic conditions (yes/no)

In the MEPS, chronic conditions can be derived from several sources, such as directly asking the respondents whether they have been diagnosed with certain priority conditions, conditions for which the respondents sought medical care, conditions that caused the participants to miss school or work or spend more than half a day in bed.^23^ We used the questions of whether the child has ever been diagnosed with asthma, the conditions for medical care were sought (diabetes, bronchitis, anxiety, depression, and bipolar disorders). Children with any of the conditions mentioned above were considered to have chronic conditions.

##### Food insecurity

MEPS special module includes specific questions about food security that are closely aligned with the United States Department of Agriculture (USDA) 10‐item scale.^24^ However, the USDA 10‐item scale queried food security in the previous 12 months, MEPS‐specific questions reference period is “previous 30 days.” Following the methodology provided by Dean et al.,^25^ we adjusted the scale for reference‐period differences. The food security questions inquire about accessing adequate food, reduced quantity, and quality of food consumption. Households that worried about food availability, households with limited food intake due to affordability, households without adequate food, and households that experienced disrupted eating were classified as having “food insecurity.” We combined the presence of chronic conditions and food insecurity to derive four categories: 1) with food insecurity and chronic conditions; 2) no food insecurity and chronic conditions; 3) with food insecurity and no chronic conditions; and 4) no food insecurity and no chronic conditions.

#### 2.3.3 Other explanatory variables

Other variables included children's age (6–11 years, 12–17 years), sex (female and male), race (White, African American, Latino, and others), and geographic locations (Northeast, South, Midwest, and West). Children's body mass index (BMI) was divided into four categories underweight, normal, overweight, and obese. These categories were determined using CDC age‐ and sex‐specific growth charts for children and teens ages 2–19.^26^ We included household income relative to the federal poverty line (poor, low‐, middle‐, and high‐income), and insurance coverage (private, public, and no insurance).

### 2.4 Statistical analysis

The analyses accounted for the complex design of the MEPS by using survey procedures with family weights. Rao‐Scott chi‐square test of independence was used to assess whether food insecurity and chronic conditions (Group 1: food insecurity with chronic conditions; Group 2: food security with chronic conditions; Group 3: food insecurity without chronic conditions; and Group 4: food security without chronic conditions) were associated with ED utilization. We calculated unadjusted and adjusted incident rate ratios (IRR) and corresponding 95% confidence intervals (CI) using Poisson regression to determine the association between food insecurity with a chronic condition and the number of ED visits. The adjustment included children's age, sex, race and ethnicity, poverty status, health insurance, and geographical location to further determine the potential confounders. These analyses were performed in SAS 9.4 and STATA 14.2.

## 3 RESULTS

There were 48.8% (unweighted *n* = 2709) female, 49.5% (unweighted *n* = 1841) White, and 50.8% (unweighted *n* = 2792) children in the age group^6^, ^7^, ^8^, ^9^, ^10^, ^11^ years. 38.7% (unweighted *n* = 2903) of children reported having FPL less than 200% (low‐income). An overwhelming majority (97%, unweighted *n* = 5334) of children had public or private health insurance. One in five children (20.9%) had a chronic condition, and 8.4% (unweighted *n* = 410) were categorized “underweight” based on BMI. Overall, 19.7% (unweighted *n* = 1399) reported having food insecurity; 9.1% (unweighted *n* = 542) visited an ED during the calendar year.

Table 1 summarizes the differences in children's characteristics by the four categories of food insecurity and chronic conditions. 6.0% of children reported both food insecurity and chronic conditions, and 13.7% reported food insecurity without chronic conditions. We found significant differences by age, race and ethnicity, poverty status, BMI categories, and region of the United States. For example, a higher percentage of children of color had food insecurity and chronic conditions (8.4% African Americans, 7.2% Hispanic, and 6.4% other races) than whites (4.7%). A higher percentage of low‐income households reported food insecurity and chronic conditions than high‐income households (15.0% vs. 1.0%). A higher percentage of children with obesity reported both food insecurity and chronic conditions (13.2% vs. 4.5%) compared to children with a healthy weight. A higher percentage of children with food insecurity and chronic conditions visited ED (19.7% vs. 13.3%) compared to the group “no food insecurity and chronic conditions.”

**Table 1:** Characteristics of school‐age children by food insecurity and chronic condition groups.

Among children aged 6–17 years											
Medical Expenditure Panel Survey, 2017											
		FI + CC		No FI + CC		FI + No CC		No FI + No CC			
		*n*	Wt%	*n*	Wt%	*n*	Wt%	*n*	Wt%	Chi‐sq	Prob
**All**		**405**	**6.0**	**773**	**14.9**	**994**	**13.7**	**3346**	**65.4**		
Sex										6.20	0.102
	Female	180	5.2	355	14.0	495	14.3	1679	66.4		
	Male	225	6.8	418	15.6	499	13.1	1667	64.5		
Race and ethnicity										90.37	<0.001
	White	117	4.7	301	16.5	191	8.7	1232	70.1		
	AA	88	8.4	163	18.5	200	18.0	482	55.1		
	Latino	163	7.2	235	10.9	517	21.2	1239	60.8		
	Other race	37	6.4	74	13.0	86	13.6	393	67.0		
Age										11.52	0.009
	6–11 Years	195	5.7	350	13.1	493	13.5	1754	67.7		
	12–17 Years	210	6.4	423	16.7	501	13.9	1592	63.0		
Region										18.68	0.028
	Northeast	64	5.0	136	18.7	119	8.5	475	67.9		
	Mid‐west	101	8.0	175	16.0	188	14.5	613	61.5		
	South	152	5.8	286	13.7	437	15.3	1324	65.1		
	West	88	5.3	176	12.8	250	14.5	934	67.5		
Poverty status										445.65	<0.001
	Poor	213	15.0	189	11.9	477	30.4	707	42.8		
	Low income	103	7.5	159	12.5	343	25.3	712	54.7		
	Middle income	72	4.7	227	15.8	142	7.7	998	71.8		
	High income	17	1.0	198	17.2	32	2.2	929	79.6		
BMI										100.12	<0.001
	Underweight	22	3.8	54	13.9	67	12.3	267	70.0		
	Normal	141	4.5	347	16.3	358	11.2	1467	67.9		
	Overweight	60	6.5	104	15.9	116	12.8	415	64.8		
	Obese	112	13.2	143	15.7	153	15.4	441	55.7		
ED Use										46.55	<0.001
	None	331	80.3	652	86.7	891	91.2	3102	92.8		
	ED Use	74	19.7	121	13.3	103	8.8	244	7.2		

*Note*: Based on 5581 children aged 6–17 years and without missing data on the food security variable. Significant differences are tested with Rao–Scott chi‐square tests. Missing data on the poverty status variable is not presented.

Abbreviations: AA, African American; BMI, Body Mass Index; CC, chronic conditions; FI, food insecurity; Wt., weighted.

The unadjusted IRR (U‐IRR) and 95% CIs from the Poisson regression of ED visits are summarized in Table 2. The unadjusted IRRs were higher for those with food insecurity and chronic conditions compared to those without food insecurity and chronic conditions (IRR = 1.99, 95% CI = 1.28–3.10 *p* < 0.001). In general, those without chronic conditions had fewer ED visits than those with chronic conditions (Table 2).

**Table 2:** Unadjusted incident rate ratio (U‐IRR) and 95% confidence intervals (CI).

From Poisson regression of number of ED visits				
Among children aged 6–17 years				
Medical Expenditure Panel Survey, 2017				
		U‐IRR	95% CI	*p*‐value
Food insecurity + Chronic condition				
	FI + CC	1.99	[1.28–3.10]	<0.001
	No FI + CC (Ref)			
	FI + No CC	0.62	[0.42–0.91]	0.014
	No FI + No CC	0.51	[0.36–0.71]	<0.001
Sex				
	Female	0.90	[0.70–1.16]	0.407
	Male (ref)			
Race and ethnicity				
	White (Ref)			
	AA	1.24	[0.88–1.75]	0.221
	Hispanic	1.01	[0.76–1.34]	0.924
	Other race	1.03	[0.65–1.65]	0.884
Age				
	6–11 years (Ref)			
	12–17 years	0.99	[0.77–1.27]	0.951
Poverty				
	Poor	1.35	[0.94–1.94]	0.107
	Low Income	1.40	[0.96–2.03]	0.080
	Middle Income	1.15	[0.77–1.71]	0.490
	High Income (ref)			
BMI				
	Underweight	0.78	[0.46–1.33]	0.359
	Normal (Ref)			
	Overweight	0.97	[0.66–1.43]	0.892
	Obese	1.11	[0.78–1.58]	0.572
Region				
	Northeast (Ref)			
	Midwest	1.60	[1.06–2.42]	0.025
	South	1.32	[0.88–1.98]	0.187
	West	1.44	[0.92–2.25]	0.106
Insurance coverage				
	Private (Ref)			
	Public	1.47	[1.15–1.97]	0.002
	Uninsured	0.48	[0.20–1.19]	0.112

*Note*: Based on 5581 children aged 6–17 years and without missing data on the food security variable. Missing data on the poverty status variable is not presented.

Abbreviations: AA, African American; BMI, Body Mass Index; CC, chronic conditions; ED, Emergency Department; FI, food insecurity; Ref, Reference Group.

When adjusted for other explanatory variables, age, sex, race and ethnicity, household poverty status, health insurance, region, and child BMI, the adjusted IRR was higher for children with chronic conditions and living in households with food insecurity (IRR = 1.90, 95% CI 1.20–3.01, *p* = 0.007). Consistent with unadjusted associations, children with no chronic conditions (with or without food insecurity) had fewer ED visits (Table 3).

**Table 3:** Adjusted incident rate ratios (A‐IRR) and 95% confidence intervals (CI).

From Poisson regression of number of ED visits				
Among children aged 6–17 years				
Medical Expenditure Panel Survey, 2017				
		A‐IRR	95% CI	*p*‐value
Food security + Chronic condition				
	FI + CC	1.90	[1.20–3.01]	0.007
	No FI + CC (Ref)			
	FI + No CC	0.61	[0.41–0.89]	0.010
	No FI + No CC	0.53	[0.37–0.75]	<0.001
Sex				
	Female	0.93	[0.73–1.20]	0.584
	Male (ref)			
Race and ethnicity				
	White (Ref)			
	AA	1.11	[0.78–1.58]	0.563
	Hispanic	0.95	[0.72–1.26]	0.737
	Other race	1.00	[0.65–1.52]	0.989
Age				
	6–11 years (Ref)			
	12–17 years	0.90	[0.70–1.15]	0.394
Poverty				
	Poor	0.81	[0.47–1.39]	0.450
	Low Income	0.99	[0.60–1.64]	0.984
	Middle Income	0.98	[0.64–1.50]	0.940
	High Income (ref)			
BMI				
	Underweight	0.80	[0.47–1.36]	0.404
	Normal (Ref)			
	Overweight	0.93	[0.63–1.39]	0.737
	Obese	0.90	[0.63–1.30]	0.581
Region				
	Northeast (Ref)			
	Midwest	1.54	[1.00–2.37]	0.051
	South	1.32	[0.85–2.03]	0.213
	West	1.46	[0.93–2.28]	0.097
Insurance coverage				
	Private (Ref)			
	Public	1.41	[0.94–2.12]	0.099
	Uninsured	0.53	[0.22–1.26]	0.151

*Note*: Based on 5581 children aged 6–17 years and without missing data on food security variable. Model adjusted for age, sex, race and ethnicity, health insurance, poverty status, region, and body mass index categories.

Abbreviations: AA, African American; BMI, Body Mass Index; CC, chronic conditions; ED, Emergency Department; FI, food insecurity; Ref, Reference Group.

## 4 DISCUSSION

In this study, we found that 1 in 16 (6.0%) children had chronic conditions and lived in households with food insecurity. Children of color, with obesity, and children living in households with a low income had a higher prevalence of food insecurity and chronic conditions (Table 1). These study findings are similar to the previous reports by Peltz and Garg. In their report, it showed that food insecurity was associated with race and ethnicity (i.e., higher food insecurity rate among African Americans and Hispanics), geographic location (i.e., Mid‐west), and low‐income household (e.g., single‐parent home and publicly insured children (compared with privately insured ones), parents unemployed), and so forth.^12^ Other studies also showed that food insecurity was associated with poverty (e.g., annual household income),^1^, ^27^ children of color (e.g., African Americans and Hispanics), and children with obesity.^1^, ^28^, ^29^ It is not surprising to find an association between food insecurity and obesity among children.^30^ Studies have found that those with food insecurity consume fewer fruits, vegetables, and protein‐rich diets^31^, ^32^ and rely more on a high‐calorie unhealthy diet,^33^ which may lead to obesity.

We also observed that children who had chronic conditions and lived in households with food insecurity had higher ED visits compared to children with chronic conditions and no food insecurity (Table 2). Even after adjusting for other potential confounding factors including socioeconomic status (poverty level and insurance coverage), personal (age, geographic location, and obesity), the relationship remained statistically significant (Table 3). Although a previous MEPS study has highlighted the role of food insecurity on healthcare use among children,^12^ the study did not analyze the interactive association of chronic conditions and food insecurity with ED use. Nevertheless, we found that food insecurity poses an additional burden among children with chronic conditions (Table 3).

Our findings raise the question of whether interventions, programs, and policies that reduce food insecurity may reduce healthcare utilization. Although there is a need for high‐quality studies on this topic,^34^ reducing food insecurity has been reported to be associated with a lower number of ED visits among dually eligible Medicaid/Medicare adults.^35^ While food security cannot cure chronic conditions, acquiring adequate food without worrying about cost can ease the financial burden on families by reducing costly acute care visits^36^ and reduce the risk of complications of chronic conditions.^37^, ^38^

Our study has strengths and limitations. We used nationally representative data with a validated food insecurity measure, which enabled us to provide insights into the needs of a specific population subgroup and potential policy interests. The results are generalizable because the data cover diverse segments of the noninstitutionalized civilian population. However, we adopted a cross‐sectional design and can only report associations. In terms of school‐age children with chronic conditions, bronchitis and anxiety certainly could present as acute disease conditions. However, MEPS did not specify chronic bronchitis or anxiety which could result in overestimations of chronic conditions in our study. Furthermore, we did not account for the bidirectional relationship between food insecurity and ED use.^39^ The food‐insecurity measure was based on the household, and we could not assess child‐specific insecurity. We also did not explore whether food‐insecure families received public assistance programs such as the Supplemental Nutrition Assistance Program (SNAP). Chronic conditions data may be incomplete because some chronic conditions were captured if they were reported as a general problem, sought medical care, missed school days, or days spent in bed.^23^

## 5 CONCLUSION

One in 16 (6.0%) school‐age children had chronic conditions and lived in households with food insecurity. Among all school‐age children with chronic conditions, children with food insecurity had 1.9 times higher ED utilization than those without food insecurity. Our findings suggest that ED visits might be leveraged to screen children at high‐risk for food insecurity.

## AUTHOR CONTRIBUTIONS

**Farheen Ghani**: Conceptualization; data curation; resources; validation; writing—original draft; writing—review & editing. **Hao Wang**: Conceptualization; formal analysis; investigation; methodology; validation; writing – original draft; writing – review & editing. **Sydney E. Manning**: Conceptualization; formal analysis; methodology; writing—review & editing. **Usha Sambamoorthi**: Conceptualization; formal analysis; methodology; resources; supervision; validation; writing—review & editing.

## CONFLICT OF INTEREST STATEMENT

Dr. Hao Wang is an Editorial Board member of Health Science Reports and a coauthor of this article. To minimize bias, he was excluded from all editorial decision‐making related to the acceptance of this article for publication.

## TRANSPARENCY STATEMENT

The lead author Hao Wang affirms that this manuscript is an honest, accurate, and transparent account of the study being reported; that no important aspects of the study have been omitted; and that any discrepancies from the study as planned (and, if relevant registered) have been explained. All authors have read and approved the final version of the manuscript. Hao Wang and Usha Sambamoorthi had full access to all the data in this study and takes complete responsibility for the integrity of the data and the accuracy of the data analysis.

## Data Availability

Data are available to the public and can be downloaded free of charge from medical expenditure panel survey download data files https://www.meps.ahrq.gov/mepsweb/data_stats/download_data_files.jsp.
